# Erosion Behavior of a Cu-Ti_3_AlC_2_ Cathode by Multi-Electric Arc

**DOI:** 10.3390/ma12182947

**Published:** 2019-09-11

**Authors:** Xiaochen Huang, Yi Feng, Liang Li, Zongqun Li

**Affiliations:** 1 School of Material and Chemical Engineering, Bengbu University, Bengbu 233030, China; liliang8008@126.com (L.L.); bbxylzq@163.com (Z.L.); 2 Engineering Technology Research Center of Silicon-based Materials, Bengbu University, Bengbu 233030, China; 3 Research and Applications of Silicon-based Materials of Bengbu University Academician Workstation, Bengbu 233030, China; 4 School of Materials Science and Engineering, Hefei University of Technology, Hefei 230009, China

**Keywords:** Cu-Ti_3_AlC_2_, cross-sectional morphology, arc erosion mechanism

## Abstract

A Cu-Ti_3_AlC_2_ cathode was eroded by arc discharging at 10 kV. The cross-sectional and horizontal morphologies of the eroded surface were recorded by a field emission scanning electron microscope (FE-SEM). The energy dispersive X-ray spectroscopy (EDS) and Raman spectrometry were carried out to analyze the compositions. The color-eroded surface was obtained by a three-dimensional laser scanning confocal microscope (3D LSCM). After 100 times of arc erosion, the Cu-Ti_3_AlC_2_ melted and resolidified. An eroded layer about 10 μm thick was formed, covered with pits, protrusions, and pores. The breakdown current was kept between 37 to 43 A. Under the action of a high temperature arc, Cu-Ti_3_AlC_2_ was oxidized to CuO and TiO_2_, accompanying the evaporation of the Al element.

## 1. Introduction

Electrical contact material is a key material for making on-off control and load current appliances in electric power and electrical circuits [1]. On top of good conductivity and thermal conductivity, electrical contact materials should also have a certain strength, hardness, weld resistance, and arc ablation resistance. Copper is widely used in electrical contacts because of its good conductivity, thermal conductivity, and low price. However, the hardness and strength of copper do not meet the use requirements, so a second phase is added to the copper matrix to improve its comprehensive performance. The typical representative of copper-based electrical contact materials was CuW. Based on the high conductivity and high strength of CuW, Liang et al. [2] focused on the effect of arc erosion on the microstructure of CuW under vacuum conditions (10^−3^ Pa). It was found that both the grain sizes of Cu and W were refined, and that the arc occurred in the specific crystal direction of Cu, while it happened randomly in W. After graphite was added into a CuW alloy, Chen et al. [3] found that the arc that originally occurred on the copper phase was transferred to the graphite, which greatly reduced the splashing phenomenon and improved the dielectric strength of the CuW alloy under vacuum conditions (10^−2^ Pa). Zhang et al. [4] found that the cathode spot moved randomly on the matrix of a copper carbon fiber composite under vacuum conditions (10^−3^ Pa), rather than on the carbon fiber. Wei et al. [5] studied the microstructures of CuCr contact alloys after 100 times of vacuum breakdown. They found that the spheres and sheets of the Cr phase appeared in the melting layer. Liu et al. [6] studied the arc ablation mechanism of Cu/Cr20 in vacuum. It was found that with the increase of current, the material began to transfer with increased arc energy. Zhang et al. [7] found that droplets, needle-like structures, pits, and bulges were formed on the eroded surface of an Al_2_O_3_-Cu/W contact material by the vacuum arc. The content of these morphologies was affected by the W content. In recent years, MAX phase materials (where M is an early transition metal, A is a group IIIA or IVA element, and X is carbon or nitrogen) are often selected as the reinforced phases of the metal matrix of contact materials because of their excellent metal and ceramic properties [8,9,10,11,12,13,14,15]. In particular, Ti_3_Al(Si)C_2_ has excellent thermal conductivity, low electrical resistivity ((2.87–3.45) × 10^−7^ Ω·m), and a low friction coefficient. In addition, the coefficient of thermal expansion of Ti_3_Al(Si)C_2_ (9.0–9.2 × 10^−6^ K^−1^) is close to that of pure Cu (8.5 × 10^−6^·K^−1^). Therefore, the outstanding properties of Ti_3_Al(Si)C_2_ make reinforcement possible for the Cu matrix to use as a potential brush and sliding electrical contact material [16,17,18]. Tian et al. [19] found that with the increase of Ti_3_AlC_2_ content, the number of surface cracks increased after arc ablation. Tungwai Leo Ngai et al. [16] studied the arc ablation characteristics of Cu-Ti_3_SiC_2_ in vacuum conditions (10^−2^ Pa). It was found that Ti_3_SiC_2_ turned into TiC_x_ after arc ablation. Previous work showed that Ti_3_AlC_2_ material had the effect of dispersing arc under atmospheric conditions, and the dispersing effect was better with higher Ti_3_AlC_2_ content [17,18,20,21]. Sun et al. [19,22,23,24,25,26] systematically studied the wettability between the Ag and MAX phases. It was found that the wettability angles between Ag and MAX were 14°, 18°, and 72°. Such good wettability significantly reduced the splashing phenomenon after arc ablation and greatly improved the anti-arc ablation performance. However, rare studies about the performance of Cu-Ti_3_AlC_2_ composites after multiple arc ablation have been previously reported. Therefore, it is necessary to understand the arc ablation mechanism of Cu-Ti_3_AlC_2_ composites for their application as a potential sliding electrical contact material. In this paper, 100 times arc ablation of Cu-20 vol.% Ti_3_AlC_2_ at 10 kV were carried out under atmospheric environment. The breakdown current was recorded after every erosion. Through characterizing the morphologies and compositions of the eroded surface and the cross-sectional sample, the arc ablation mechanism of the Cu-Ti_3_AlC_2_ composite was systematically explained. 

## 2. Materials and Methods

Copper powder (99.5%, ≤45 μm, Sinopharm Chemical Reagent Co., Ltd. Shanghai, China) and Ti_3_AlC_2_ powder (99.2%, ~5 μm, for details on synthesis process, see [27]) were mixed for 3 h with a constant Cu/Ti_3_AlC_2_ volume ratio of 80:20. The mixture was put into a high strength graphite mold. Subsequently, under a pressure of 30 MPa, the mixture was held at 800 °C for 1 h, surrounded by an Ar atmosphere in a hot-pressing furnace (ZT-40-20Y, Shanghai Chenhua Science Technology Corp., Ltd, Shanghai, China). Then, the mold was cooled down in the furnace. The polished Cu-20 vol.% Ti_3_AlC_2_ was chosen as the cathode, which can be moved upward and downward by a stepping motor controller (AKS-01Z, Shanghai Siheng Motor Manufacturing Co., Ltd., Shanghai, China). A W rod was chosen as the anode. The load voltage of 10 kV was added between the cathode and anode. The cathode moved towards the anode with a velocity of 0.2 mm/min until the electric arc was ignited. Once the arc was generated between the electrodes, the breakdown current was recorded by the digital memory oscilloscope (ADS1102CAL, Shenzhen Anxintai Electronics Co., Ltd, Shenzhen, China). The experimental procedures of the arc discharging test were described in [18]. After the arc discharging test was repeated 100 times in air atmosphere, the morphology of the eroded surface was recorded by a field emission scanning electron microscope with energy dispersive X-ray spectroscopy (FE-SEM, JEM-2100F, JEOL, Tokyo, Japan) system. Three-dimensional laser scanning with a confocal microscope (3D LSCM, VK-X1000, Keyence, Osaka, Japan) was conducted to reconstruct the morphology characteristics with laser color observations. The cross-sectional images of the eroded surfaces were recorded by scanning electron microscopy (SEM, Tabletop microscopes TM4000Plus, Hitachi, Tokyo, Japan) with the image signals of backscattered electrons. The products of the eroded samples were analyzed by a Raman spectrometer (LabRAM HR, HORIBA JOBIN YVON, Paris, France), which was performed over a wavenumber range of 100–1000 cm^−1^ with an Nd: YAG laser.

## 3. Results and Discussion

**Figure 1:**
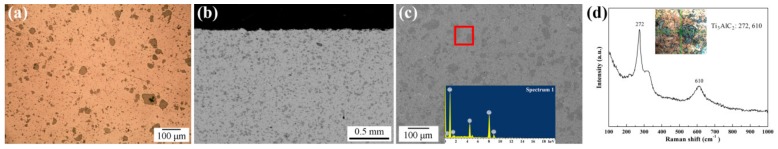
Original surface morphologies of Cu-20 vol.% Ti_3_AlC_2_ material by (**a**) 3D LSCM, (**c**) SEM, (**d**) Raman spectrometer. (**b**) Cross-sectional image of original Cu-20 vol.% Ti_3_AlC_2_ surface.

In order to compare the samples before and after arc erosion, the corresponding pictures of the original Cu-20 vol.% Ti_3_AlC_2_ material are shown in Figure 1. Figure 1a was obtained by 3D LSCM with laser color observation. Figure 1b,c were obtained by SEM. Figure 1b is the cross-sectional image. Spectrum 1 mainly contains Cu, Ti, and Al elements. The data are listed in Table 1. The laser of the Raman spectrometer is shown on the green cross in the inset of Figure 1d, where a gray particle is located. The peaks of 272 and 610 cm^−1^ corresponded to Ti_3_AlC_2_ [28]. It can be seen from Figure 1a–c that Ti_3_AlC_2_ particles were evenly distributed on the copper matrix. 

**Figure 2:**
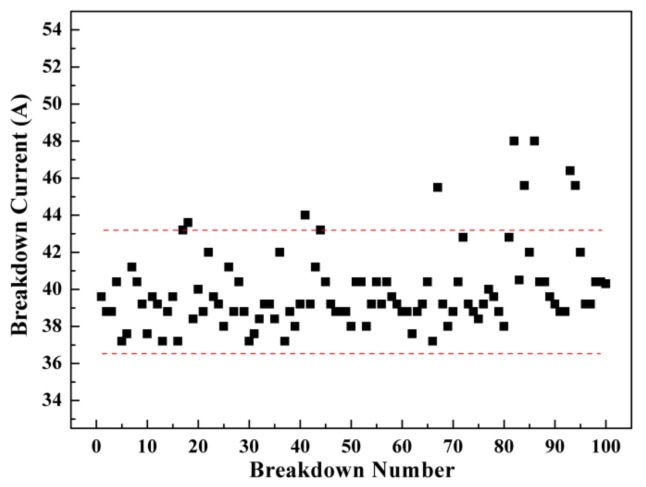
Curve of breakdown current to breakdown number.

After each arc discharging test, the breakdown current was recorded, as shown in Figure 2. It can be seen from the figure that the breakdown current is basically between 37 and 43 A. It is worth noting that 10% of the breakdown current data points fell in the 43 to 48 A range. The results indicate that the breakdown current does not change much with the increase of arc discharge times. For a certain material, if the current between the cathode and anode increases, the friction coefficient and wear rate of the sliding electrical contact material will seriously increase, resulting in the seriously wear and premature failure of the material [29]. The stable breakdown current of Cu-20 vol.% Ti_3_AlC_2_ has little influence on the wear properties of sliding electrical contact materials.

**Figure 3:**
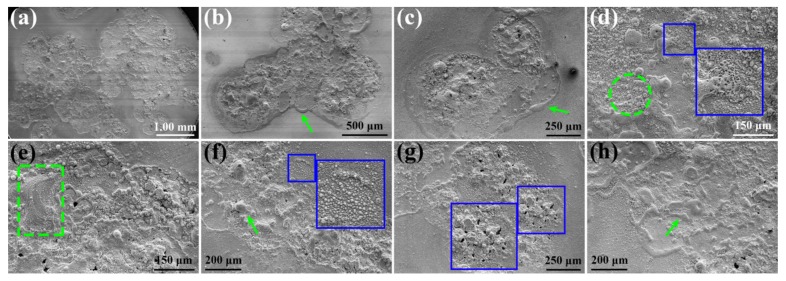
SEM images of (**a**) whole eroded Cu-Ti_3_AlC_2_ surface; (**b**,**c**) ablation edge; (**d**) coral-like particles; (**e**) molten liquid accumulation; (**f**) protrusions; (**g**) black holes; (**h**) ablation edge after being eroded 100 times by arc discharging.

Micro-morphologies of the eroded Cu-Ti_3_AlC_2_ are displayed in Figure 3. Figure 3a is the whole surface of the eroded Cu-Ti_3_AlC_2_ cathode, covered with pits, protrusions, and pores. Under the high energy of the electric arc, the Cu-Ti_3_AlC_2_ composite melts and resolidifies, leaving the morphologies of ablation edge on the surface, as shown by green arrows in Figure 3b,c,h, which is an example of one of the 100 times of arc discharging. In Figure 3d, coral-like particles are formed in the green circle. The bigger blue rectangle in Figure 3d is the enlarged image of the smaller blue rectangle. Obviously, pores are generated on the eroded surface, which is caused by the changed solubility of gases in air during the rapid solidification process [30]. Under the action of electromagnetic force, gravity, and plasma force, the molten liquid accumulates in one direction, as shown in the green rectangle in Figure 3e. Some eroded area accumulates to small protrusions, as indicated by the arrow in Figure 3f. In the magnified image in the blue rectangle in Figure 3f, sputtered particles are observed, which also arises from the forces mentioned above. Black holes are revealed in the blue rectangle in Figure 3g, which indicates that the material evaporates into the surroundings.

**Figure 4:**
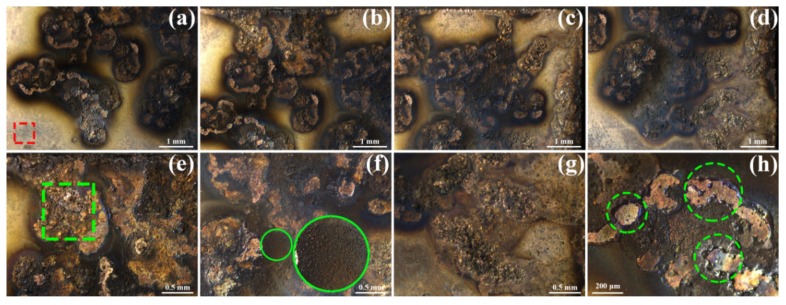
3D LSCM images of (**a**)–(**d**) whole eroded Cu-Ti_3_AlC_2_ surface; (**e**) and (**g**) morphologies caused by forces; (**f**) sputtered particles; (**h**) magnified morphology in (**b**).

The images obtained by 3D LSCM with laser color observation are close to the original appearance of the material, which are shown in Figure 4. In Figure 4a, the area in red rectangle is the original material. It can be seen from Figure 4a–d that the eroded surfaces are black. Some areas are yellower than the original area, which is always surrounded by a black area. These morphologies are magnified in green circles in Figure 4h. The yellower areas correspond to the eroded center. Under the action of the electric arc, the material melts and moves around, resulting in the exposure of the copper matrix, as seen in the green circles in Figure 4h. Meanwhile, the resolidified material forms the color edges. Under the action of electromagnetic force, gravity, and plasma force, small particles are sputtered on the eroded surface, as seen in the green circles in Figure 4f. The protrusions in Figure 4g and the area in the green rectangle in Figure 4e also arise from these forces.

**Figure 5:**
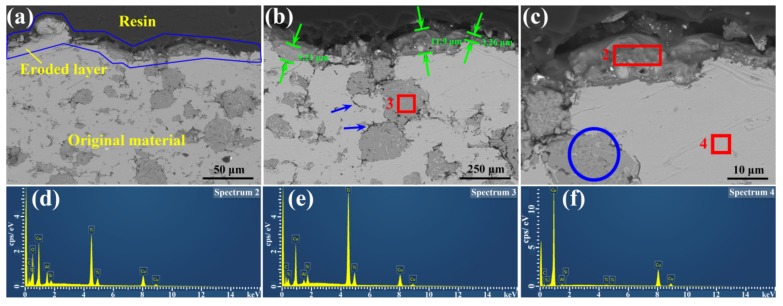
(**a**)–(**c**) Cross-sectional image of Cu-Ti_3_AlC_2_ surface after being eroded 100 times by arc discharging; Energy dispersive X-ray spectroscopy (EDS) results of (**d**) in the rectangle in (**c**); (**e**) in the rectangle in (**b**) and (**f**) in the rectangle in (**c**).

In order to observe the changes of longitudinal microstructures and compositions of materials, cross-sectional images are shown in Figure 5. Figure 5a–c were obtained with the signals of backscattered electrons, which can display the distribution of elements and phases. It can be seen from Figure 5a that the image is divided into three regions. The region surrounded by blue lines is the eroded layer. On the top of the layer is resin, which comes from the sample preparation. The remaining region is the original material, which contains two colors, gray and dark gray. The eroded layer is about 10 μm thick, as labeled in Figure 5b. Under the eroded layer, micro cracks are formed along grain boundaries, as shown in blue arrows in Figure 5b. In order to preliminarily analyze the distribution of elements on the eroded Cu-Ti_3_AlC_2_ surface, energy dispersive X-ray spectroscopy (EDS) was used in the areas in red rectangles in Figure 5b,c. Spectrum 2 was obtained as seen in red rectangle 2 in Figure 5c, which mainly contains O element, indicating that the material is oxidized under the action of electric arc. The data are listed in Table 1. The types of oxides still need to be further confirmed by Raman spectroscopy at a later time. It is worth noting that the value of Ti/Al is bigger than the theoretical value 3, meaning that part of the Al elements escape from Ti_3_AlC_2_, due to the weak combination with Ti and C elements [26]. Spectrum 3 and spectrum 4 were obtained from the dark gray area and gray areas in Figure 5b,c, respectively, as shown specifically in Figure 5e,f. The dark gray area in the red rectangle in Figure 5b mainly contains Ti element, indicating that the area is Ti_3_AlC_2_. However, the value of Ti/Al is larger than 3, meaning that some Al elements diffuse out to the Cu matrix. Meanwhile, Cu elements diffuse into the Ti_3_AlC_2_ crystal structure, resulting in a minor amount of Cu element also being detected. The gray area in red rectangle 4 in Figure 5c mainly contains Cu element, while minor amounts of Al element are also detected, which arises from the diffusion of Al from Ti_3_AlC_2_ [31]. Minor Si elements are detected from spectrum 2 to 4, which arise from the abrasive papers during the polishing process.

**Figure 6:**
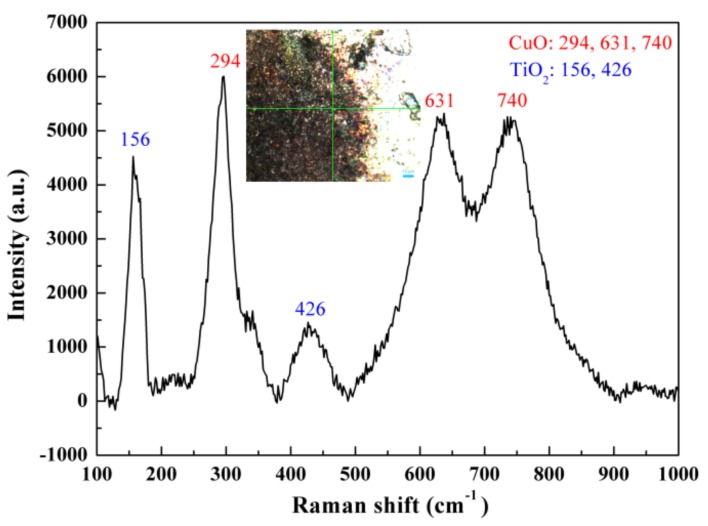
Raman spectrum of Cu-Ti_3_AlC_2_ surface after being eroded 100 times by arc discharging.

In order to confirm the products on the eroded surface of Cu-Ti_3_AlC_2_, the Raman spectrum is shown in Figure 6. The laser acts at the green cross. The peaks at 294, 631, and 740 cm^−1^ are assigned to CuO (R060978, R120076). The peaks located at 156 and 426 cm^−1^ arise from TiO_2_ [32,33,34]. Raman spectrum results demonstrate that the Cu-Ti_3_AlC_2_ cathode is oxidized to TiO_2_ and CuO. Aluminum oxide is not detected, possibly because its content is very low, and there’s no aluminum oxide generated on the area of laser action. The peak intensity of the generated CuO is about 6000 a.u. or 5500 a.u., which is higher than that of TiO_2_ (4500 a.u. or 1500 a.u.), indicating that the copper is oxidized more quantitatively than Ti [35,36,37].

**Figure 7:**
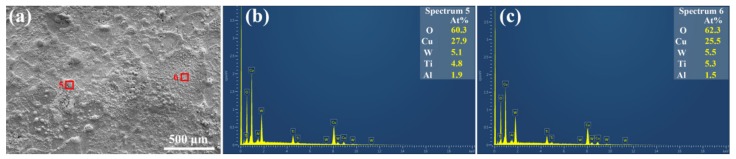
(**a**) SEM morphology of the eroded Cu-Ti_3_AlC_2_ surface by 100 times arc discharging; EDS results in (**b**) and (**c**) corresponding to the rectangles 5 and 6 in (**a**), respectively.

Except for the component detection from the cross-sectional eroded layer, the results of which are also revealed in the horizontal direction in Figure 7. Spectrums 5 and 6 in Figure 7b,c are detected in the areas of rectangles 5 and 6 in Figure 7a, respectively. The data are listed in lines 6 and 7 in Table 1. Both spectrum 5 and 6 mainly contain O element. If all the detected Cu, Ti, Al, and W are oxidized to CuO, TiO_2_, Al_2_O_3_, and WO_2_, the atomic percentages of O element should be 50.55 and 49.35, which are lower than the detected values of 60.3 and 62.3, respectively. In Figure 3d, pores are formed on the eroded surface. During the process of arc ablation, gases are dissolved in the material, some gases escape, and some gases remain in the resolidified material, which results in the higher O content in spectrums 5 and 6. It is worth noting that a minor amount of W element is also detected, which comes from the W anode, due to the multi-electric arc. What’s more, previous studies have shown that the addition of a single metal oxide in electric contact materials can effectively improve the wettability between the matrix material and the oxide, reducing both the droplet splashes and the mass loss of a cathode material [38,39,40]. The effect of TiO_2_ and CuO on the wettability of Cu-Ti_3_AlC_2_ still needs further systematic study in the future.

## 4. Conclusions

In the application process of electrical contact materials, arcs often appear. Here, a Cu-Ti_3_AlC_2_ cathode, a potential electrical contact material, is eroded by arc discharging at a load voltage of 10 kV for 100 times in air. The breakdown current is kept between 37 and 43 A and does not change significantly with the increase of breakdown times, which has little influence on the wear properties of sliding electrical contact materials. Under the action of the electric arc, pits and protrusions are formed on the eroded surface. The material melts and resolidifies with a colored edge. The eroded layer is about 10 μm thick, as detected from the cross-sectional image. Cu-Ti_3_AlC_2_ is oxidized to CuO and TiO_2_, which may improve the wettability between the matrix material and its reinforcement. Meanwhile, aluminum oxides evaporate to the surrounding area and the Al diffuses out to the Cu matrix. Due to the multi-electric arc, the W element on the anode is transferred to the Cu-Ti_3_AlC_2_ cathode. Due to the stable breakdown current and slight sputtered particles, Cu-Ti_3_AlC_2_ is more suitable than a Cu or Cu alloy for sliding electrical contact material. This study is of great significance to the application of Cu-Ti_3_AlC_2_ material as a sliding contact material, and lays a foundation for further research on electrical friction and wear properties under the same environmental conditions.

## Figures and Tables

**Table 1:** The energy dispersive X-ray spectroscopy (EDS) results from Figure 5 and Figure 7.

Spectrum	Cu	O	Ti	Al	Si	W	Ti/Al
1	75.67	0	19.99	4.34	0	0	>3
2	12.81	49.28	14.98	2.87	0.52	0	>3
3	18.35	0	38.79	1.32	2.39	0	>3
4	66.47	0	0.67	1.85	0.56	0	<3
5	27.9	60.3	4.8	1.9	–	5.1	<3
6	25.5	62.3	5.3	1.5	–	5.5	>3
